# Molecular and Cellular Mechanisms Linking Mood Disorders, HPA Axis Dysregulation, and Neurocognitive Inflammation to Perioperative Neurocognitive Disorders

**DOI:** 10.3390/biom16081179

**Published:** 2026-08-12

**Authors:** Alyson Sato, Nicole Chang, Nebojsa Nick Knezevic, Chanannait Paisansathan

**Affiliations:** 1 University of Illinois College of Medicine, Chicago Campus, Chicago, IL 60612, USA; asato4@uic.edu (A.S.); nchan36@uic.edu (N.C.); 2 Department of Anesthesiology, University of Illinois, Chicago, IL 60612, USA; nebojsa.knezevic@aah.org; 3 Department of Anesthesiology, Advocate Illinois Masonic, Chicago, IL 60657, USA; 4 Department of Surgery, University of Illinois, Chicago, IL 60612, USA; 5 Department of Anesthesia and Critical Care, University of Chicago, Chicago, IL 60637, USA

**Keywords:** perioperative neurocognitive disorders, mood disorders, hypothalamic–pituitary–adrenal axis, chronic neuroinflammation

## Abstract

Perioperative neurocognitive disorders (PND) encompass a spectrum of cognitive impairments occurring across the surgical period and are associated with significant morbidity, delayed recovery, and reduced quality of life. Although established risk factors include advanced age, cardiovascular disease, and preexisting cognitive impairment, the contribution of mood disorders to PND susceptibility remains incompletely understood. This review systematically examines the neurobiological overlap between mood disorders, particularly major depressive disorder (MDD) and bipolar disorder, and PND, with emphasis on shared biomolecular mechanisms. We identify convergent pathophysiologic pathways including hypothalamic–pituitary–adrenal (HPA) axis dysregulation, chronic neuroinflammation, NF-κB-mediated cytokine signaling, microglial priming, tryptophan–kynurenine pathway dysregulation, and brain-derived neurotrophic factor (BDNF) suppression. These mechanisms collectively suggest that patients with preexisting mood disorders may enter surgery in a biologically sensitized neuroimmune state, lowering the threshold for exaggerated neuroinflammatory responses and postoperative cognitive dysfunction. Recognition of mood disorders as modifiable perioperative vulnerability states may inform preoperative risk stratification, guide anesthetic and analgesic management, and support the development of targeted interventions to reduce postoperative cognitive complications and improve surgical outcomes.

## 1. Introduction

**Figure 1:**
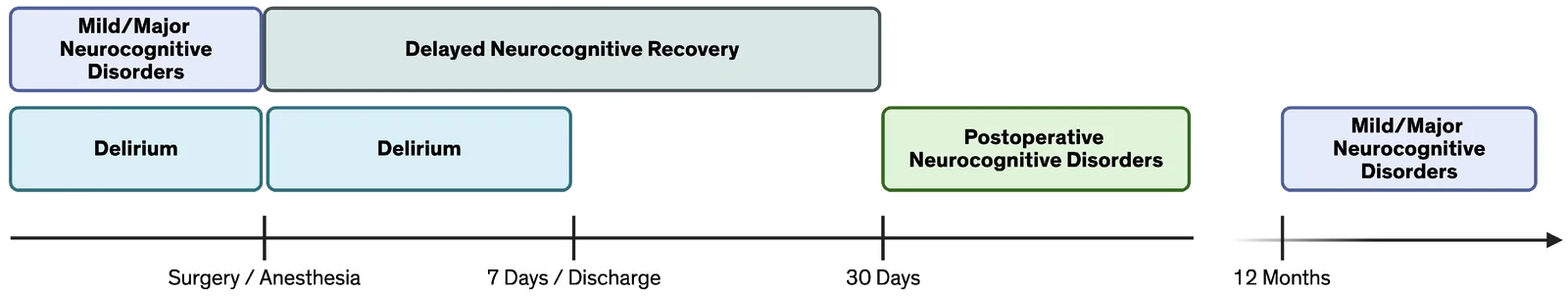
Classification of perioperative neurocognitive disorders. Created in BioRender https://BioRender.com/pkvn3a5, accessed on 27 July 2026.

Perioperative neurocognitive disorders (PNDs) include a spectrum of cognitive impairments identified across the perioperative period defined by the timing and duration (Figure 1) [1]. PNDs range from preexisting cognitive impairment and postoperative delirium to delayed neurocognitive recovery within 30 days and postoperative cognitive dysfunction (POCD), which can persist for up to 12 months after surgery. These conditions are associated with prolonged hospitalization, delayed functional recovery, loss of independence, and increased morbidity and mortality [2,3,4]. Despite their clinical significance, the mechanisms underlying PNDs remain incompletely understood. Emerging evidence points to perioperative stress responses (including neuroinflammatory and neuroendocrine dysregulation) as central contributors to cognitive vulnerability [5,6]. In particular, mood disorders may induce a state of neuroimmune priming characterized by chronic microglial sensitization, astrocyte activation, hypothalamic–pituitary–adrenal (HPA) axis dysregulation, and persistent low-grade inflammation. In this setting, acute perioperative stressors may provoke an exaggerated neuroinflammatory response, increasing vulnerability to PND. Understanding the overlap between perioperative inflammatory signaling and the neurobiological mechanisms underlying mood disorders may therefore provide important insight into patient susceptibility, risk stratification, and perioperative optimization.

## 2. Epidemiology and Risk Factors of Perioperative Neurocognitive Disorders

Perioperative neurocognitive disorders (PNDs) are common complications following surgery, particularly among older adults. Preexisting cognitive impairment is present in approximately 18% of elective surgical patients aged ≥65 years, with higher rates identified through formal cognitive testing [7]. Postoperative delirium occurs in approximately 2.5–4.5% of the general surgical population, but incidence increases substantially in older adults and patients undergoing cardiac surgery, where rates may approach 46% [8,9,10,11]. POCD affects both older and younger populations, occurring in approximately 36% of younger and 42% of older patients at hospital discharge, with persistent deficits at 3 months observed in up to 5% and 13%, respectively [12,13,14].

Multiple patient-level risk factors contribute to PND susceptibility, including advanced age, lower educational attainment, preexisting cognitive impairment, and neurologic or cerebrovascular disease [15,16,17,18,19]. Surgical factors also influence risk, with cardiac procedures, perioperative complications, and heightened physiologic stress responses associated with increased incidence of postoperative delirium and cognitive dysfunction [10,11].

Emerging evidence suggests that preoperative mood disorders, particularly major depressive disorder (MDD), may increase susceptibility to postoperative delirium and longer-term cognitive decline [20,21,22]. Unlike many established risk factors, mood disorders may represent potentially modifiable contributors to PND vulnerability [23]. The established associations between mood disorders, chronic neuroinflammation, and hypothalamic–pituitary–adrenal (HPA) axis dysregulation underlie the development of exaggerated perioperative neuroimmune responses and cognitive dysfunction.

## 3. Mechanistic Pathways Linking Mood Disorders and PNDs

### 3.1. Surgical Trauma and Neuroinflammation

Surgical trauma induces a complex systemic inflammatory response that is increasingly recognized as a central mechanism underlying PNDs. Tissue injury during surgery releases damage-associated molecular patterns (DAMPs), endogenous molecules from stressed or injured cells that trigger sterile inflammatory signaling [24]. Examples of DAMPs include S100 proteins, Aβ, HMGB1, and heat shock proteins. Although these molecules play crucial roles in normal gene activation, cell proliferation and innate immune signaling, excessive DAMP release may promote maladaptive neuroinflammatory responses.

**Figure 2:**
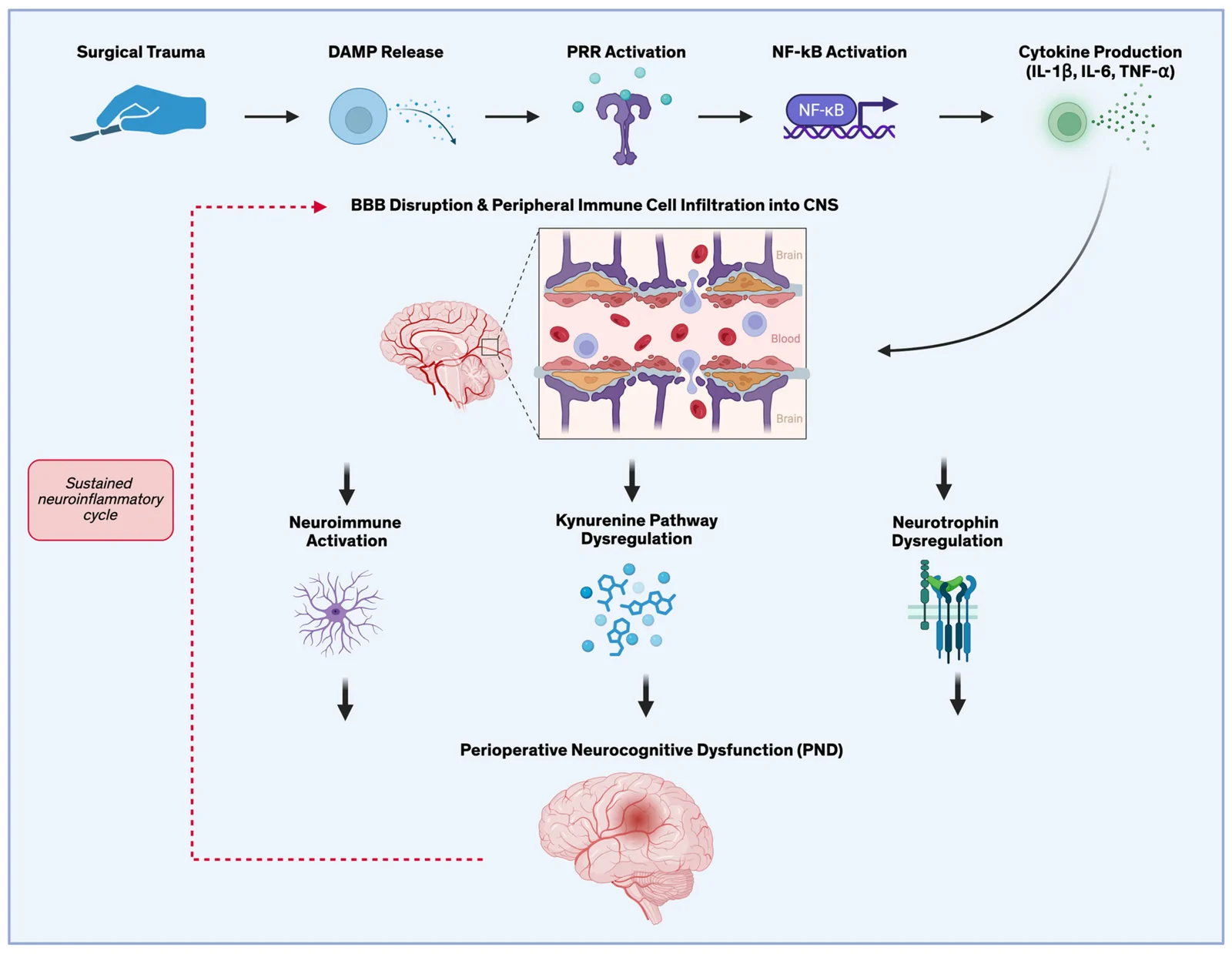
Neuroinflammatory mechanisms underlying perioperative neurocognitive dysfunction. Created in BioRender https://BioRender.com/2o3pyxw, accessed on 5 August 2026.

Following peripheral tissue injury, DAMPs accumulate and activate pattern-recognition receptors (PRRs) expressed on peripheral immune cells and microglia, including toll-like receptors (i.e., TLR4 and TLR1/2), NOD-like receptors (i.e., NLRP3 inflammasome), and C-type lectin receptors (i.e., CLEC7A) (Figure 2) [25]. Activation of these receptors induces intracellular signaling cascades involving inflammasome assembly, caspase-1 activation and nuclear factor kappa B (NF-κB), ultimately resulting in secretion of proinflammatory cytokines including interleukin-1β (IL-1β), interleukin-6 (IL-6), and tumor necrosis factor-α (TNF-α) [24,26,27].

NF-κB is a family of inducible transcription factors that regulate immune and inflammatory responses [28]. There are two major pathways that activate NF-κB: the canonical and noncanonical pathways. The canonical pathway is mainly involved in stress response, acute inflammation and innate immunity. It is a more rapid process with a wide array of triggers and signals, including cytokines, PRRS, and TNF-α. In contrast, the noncanonical pathway plays a role in lymphoid organ development and adaptive immunity [29]. This process is slower, with more selective responses to specific stimuli, including ligands of TNF receptors (TNFRs).

Through the canonical NF-κB pathway, activation of TNFRs, toll-like receptors (TLRs) and other PRRs initiates intracellular signaling cascades that result in degradation of IkB, allowing NF-κB dimers to translocate to the nucleus and induce transcription of proinflammatory target genes [29,30]. Target genes include interleukins, Fas and Bcl-2, among others. These proteins promote cell survival [30].

Dysregulation of NF-κB is a core aspect of acute and chronic inflammation, leading to upregulation of proinflammatory pathways through cytokine release. These cytokines contribute to systemic inflammation and to the disruption of blood–brain barrier (BBB) integrity, thereby facilitating peripheral-to-central immune signaling [27].

Compromise of the BBB permits inflammatory mediators and peripheral immune cells, including bone marrow-derived monocytes (BM-DMs), to access the CNS (Figure 2) [27]. Within the CNS, these inflammatory signals promote activation of resident microglia and amplification of local cytokine production and complement cascade activation [31]. Activated microglia transition from a surveillant ‘resting’ M0 microglial state toward a proinflammatory M1 microglial phenotype characterized by increased cytokine release, oxidative stress, and neurotoxic signaling [26,32]. Several cytokines that are released, including IFN-α/β, IFN-γ, TNF-α, IL-1β, IL-10, and TGF-β, have been implicated in regulating the activation and phenotypic polarization of quiescent microglia [33].

While microglia serve as CNS phagocytes and play key roles in neuroinflammation, other glial cell types may also contribute to the development of PNDs [31]. Astrocytes, the most abundant glial cell type in the CNS, are essential for maintaining BBB integrity, regulating synaptic function and providing metabolic support to neurons [34]. In response to microglial activation, astrocytes can adopt reactive phenotypes that amplify neuroinflammatory signaling through the release of proinflammatory cytokines such as IL-6, TNF-α and IL-1β. Moreover, reactive astrocytes can undergo astrogliosis in response to inflammatory signals, leading to fibrotic tissue formation to stop the spread of damaged or inflammatory tissues [31]. At the same time, reactive astrocytes may amplify neuroinflammation through the release of proinflammatory mediators that sustain and further activate microglia.

The bidirectional relationship between microglia and astrocytes is increasingly recognized as a central component of neuroinflammation. Astrocytes respond to microglial complement cascades, C5, C3 and C1q, and further perpetuate inflammation in the CNS through release of additional cytokines, chemokines and reactive oxidative species (ROS), sustaining microglial activation [35]. This cycle may contribute to prolonged inflammatory signaling, synaptic dysfunction, and CNS injury. Perioperatively, chronic stress exposure leading to preexisting glial activation may therefore create a biologically sensitized state in which acute perioperative stressors trigger exaggerated neuroimmune responses and increase vulnerability to PND.

In addition to inflammatory signaling, activated microglia and reactive astrocytes may contribute to disruptions in neuronal signaling and synaptic function. Under physiologic conditions, astrocytes support synaptic plasticity through calcium-dependent signaling pathways and the release of neurotransmitters, neurotrophic factors, and metabolic substrates that regulate neuronal activity [36]. During neuroinflammatory states, these homeostatic functions may become impaired, contributing to neuronal dysfunction. Recent evidence suggests that Orai1-mediated calcium signaling serves as a critical regulator of microglial state transitions, promoting proinflammatory gene expression, astrocyte activation, and neuroinflammation-driven behavioral changes [37]. These findings further support the role of microglial–astrocytic crosstalk as a central mechanism linking neuroinflammation to cognitive dysfunction.

Downstream neuroinflammatory signaling disrupts neurotransmitter homeostasis, impairs synaptic plasticity, and alters excitatory–inhibitory balance within regions especially important for cognition, particularly the hippocampus. Unlike other brain macrophages, microglia exhibit remarkable morphological and functional plasticity, dynamically shifting between a surveillant (M0) state and activated phenotypes (M1 and M2) in response to environmental cues [25]. In their homeostatic M0 state, microglia continuously extend and retract highly motile processes to monitor the CNS microenvironment and maintain neural circuit integrity. Activation toward the proinflammatory M1 phenotype promotes complement-mediated synaptic pruning and disruption of long-term potentiation, contributing to impaired synaptic connectivity and deficits in learning and memory [38]. M1 microglia further contribute to neuronal injury and apoptosis through the release of reactive oxygen and nitrogen species, while chronic neuroinflammation further impairs hippocampal neurogenesis [25,39]. Collectively, these mechanisms converge to produce neuronal dysfunction and cognitive vulnerability following surgery (Figure 2).

Although perioperative inflammation may occur in all surgical patients, preexisting neuroimmune dysregulation may amplify susceptibility to maladaptive inflammatory responses. Chronic stress, mood disorders, and hypothalamic–pituitary–adrenal (HPA) axis dysfunction have all been associated with persistent inflammatory signaling and microglial sensitization, suggesting that some patients may enter surgery in a biologically primed neuroinflammatory state that increases vulnerability to PND.

### 3.2. Microglial Priming and Synaptic Dysfunction in PNDs and Mood Disorders

Microglia are the resident immune cells of the CNS and are essential for maintenance of neuronal homeostasis, synaptic remodeling, and neuroimmune surveillance [40]. They exhibit remarkable morphological and functional plasticity, transitioning between homeostatic and activated phenotypes in response to environmental cues. Beyond their immunologic functions, microglia are highly integrated into neuronal signaling networks and play critical roles in synaptic plasticity, neurotransmission, and neural circuit maintenance. Microglia express receptors for multiple neurotransmitters and neuropeptides, including glutamate, γ-aminobutyric acid (GABA), dopamine, serotonin, and norepinephrine, allowing them to respond dynamically to changes in neuronal activity [25]. Through the release of cytokines, reactive oxygen species (ROS), complement proteins, and neurotrophic factors, microglia regulate dendritic spine remodeling, synaptic pruning, neuronal excitability, and long-term synaptic plasticity [41].

Although transient microglial activation may support tissue repair and restoration of homeostasis, persistent or dysregulated activation can promote maladaptive neuroinflammation and synaptic dysfunction [41]. Chronic neuroinflammatory signaling and excessive complement-mediated synaptic pruning have been increasingly implicated not only in neurodegenerative disease and PNDs, but also in mood disorders such as depression and anxiety [42,43]. Elevated inflammatory cytokines may alter monoaminergic signaling, impair hippocampal neurogenesis, and disrupt limbic and cortical circuitry involved in emotional regulation, suggesting substantial mechanistic overlap between mood disorders and perioperative neuroinflammation [44].

Stress-induced microglial dysfunction further drives this neuroimmune vulnerability. Chronic stress has been associated with dendritic atrophy and spine loss within the medial prefrontal cortex (mPFC), followed by compensatory dendritic remodeling during stress recovery [45]. In parallel, stress-induced changes in microglial morphology have been linked to altered CX3CL1-CX3CR1 signaling, a pathway involved in neuron–microglia communication and regulation of microglial activation, as well as CSF1-CSF1R signaling, which influences microglial proliferation and survival [46]. Experimental models have demonstrated that mice lacking CX3CR1 exhibit reduced microglial phagocytic capacity and attenuation of stress-induced synaptic plasticity deficits within the hippocampus [47]. Through synaptic pruning, engulfment of dendritic elements, direct neuron–microglia interactions, and secretion of neurotrophic factors, microglia contribute to both adaptive and maladaptive neuronal remodeling following stress exposure.

Collectively, these findings indicate that chronic stress and mood disorders establish a primed microglial state defined by persistent neuroinflammatory sensitization and altered synaptic regulation. Consequently, acute perioperative inflammatory stressors trigger exaggerated neuroimmune activation, driving susceptibility to postoperative cognitive impairment.

### 3.3. The Role of Astrocytes in Mood Disorders

Emerging molecular evidence has shifted the understanding of major depressive disorder (MDD) and bipolar disorder away from a simple monoaminergic deficit toward a complex model of glial dysfunction, wherein astrocytic pathology serves as a primary driver of mood dysregulation. Postmortem tissue analyses from depressed individuals consistently demonstrate a localized reduction in the density and overall number of glial fibrillary acidic protein (GFAP)-positive astrocytes, particularly within fronto-limbic circuits such as the dorsolateral prefrontal cortex and the hippocampus [48,49]. This structural atrophy leads to profound homeostatic failures at the tripartite synapse [50]. Crucially, the downregulation of astrocytic glutamate transporters—specifically GLT-1 and GLAST—disrupts normal glutamate–glutamine cycling, allowing excess neurotransmitter to persist within the synaptic cleft [51,52]. This accumulation induces low-grade, widespread glutamatergic excitotoxicity at neuronal NMDA receptors, resulting in accelerated synaptic pruning and impaired long-term potentiation [53,54].

Furthermore, chronic stress impairs astrocytic metabolic support by downregulating glucose transporters and inhibiting lactate dehydrogenase A (LDHA) [55]. This dismantling of the critical astrocyte–neuron lactate shuttle deprives high-energy cognitive circuits of their essential energetic substrates, contributing to the localized parenchymal hypometabolism frequently visualized in functional neuroimaging of depressed patients [54,56,57]. Combined with a chronic reduction in astrocytic neurotrophic support—namely brain-derived neurotrophic factor (BDNF) and pleiotrophin (PTN)—and the fragmentation of gap junction networks, the central nervous system is left structurally and metabolically vulnerable. Connexins are transmembrane proteins that form gap junctions and hemichannels, which regulate direct intercellular communication throughout the central nervous system. Consequently, dysfunction or disruption of these gap junction networks impairs intercellular communication, compromising astrocytic syncytial integrity and homeostatic support. Specifically, alterations in Cx43 expression and function, alongside its accelerated degradation—the primary astrocytic connexin in the prefrontal, hippocampal, and orbitofrontal regions—fuel a vicious cycle of sustained neuroinflammation in animal models of depression [58,59,60,61]. Ultimately, this baseline glial frailty destabilizes neural circuit plasticity and desynchronizes top-down emotional regulation, cementing the persistent affective and cognitive impairments that characterize severe mood disorders [54].

### 3.4. Hypothalamic–Pituitary–Adrenal Axis Dysregulation and Stress Sensitization

**Figure 3:**
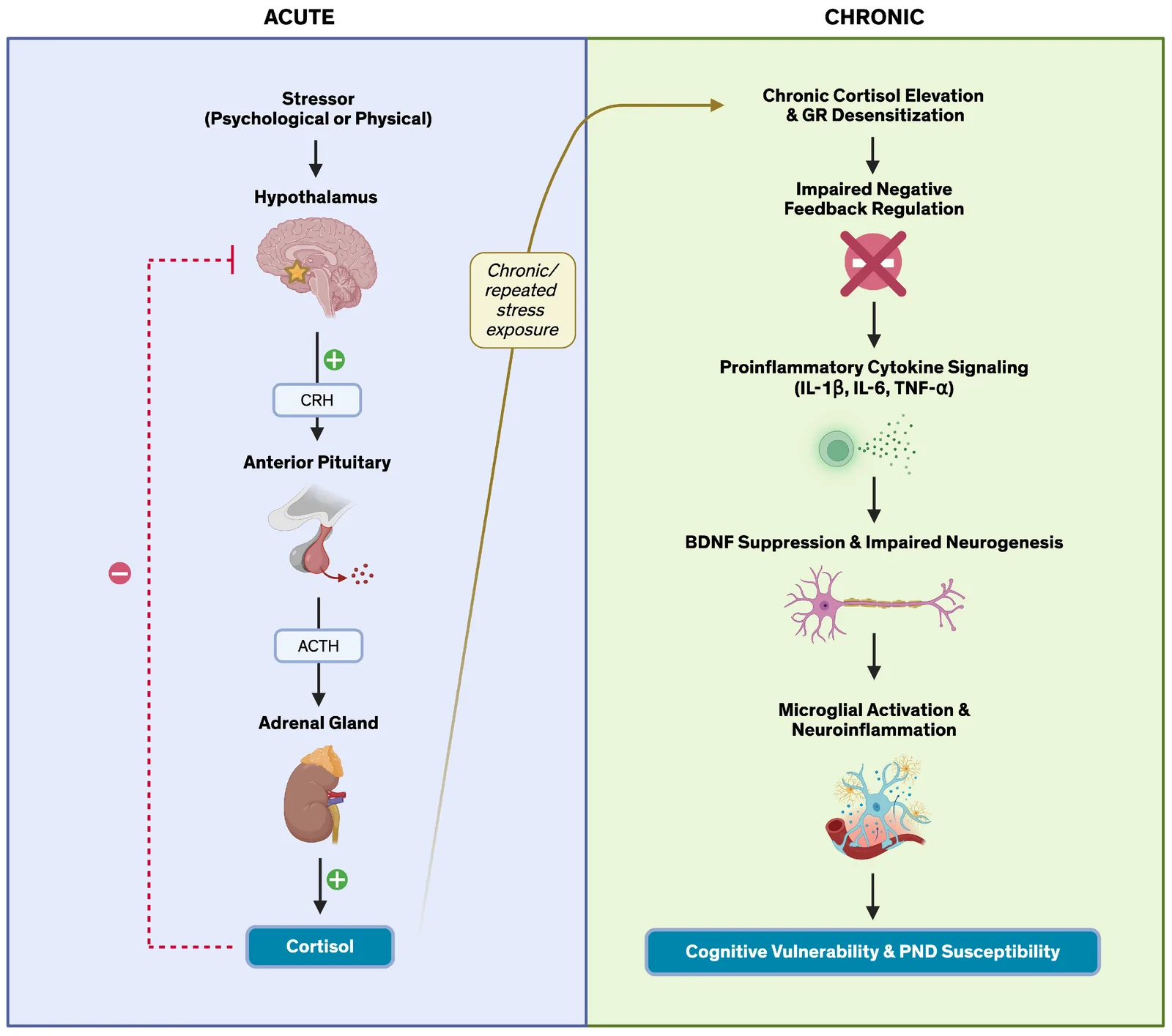
The hypothalamic–pituitary–adrenal axis and downstream consequences of chronic cortisol elevation on perioperative neurocognitive vulnerability. Created in BioRender https://BioRender.com/cia59m9, accessed on 25 June 2026.

The hypothalamic–pituitary–adrenal (HPA) axis is a crucial part of the neuroendocrine system responsible for coordinating physiologic responses to psychological and physical stressors while maintaining systemic homeostasis, commonly referred to as the ‘fight-or-flight’ response. This complex neuroendocrine system is regulated by the suprachiasmatic nucleus (SCN), which coordinates circadian rhythm and activation of the HPA axis, ultimately leading to cortisol secretion from the adrenal cortex (Figure 3) [62,63].

In response to physiologic or psychological stress, the hypothalamus releases corticotropin-releasing hormone (CRH), which stimulates the anterior pituitary to secrete adrenocorticotropic hormone (ACTH) (Figure 3). ACTH subsequently acts on the adrenal cortex to promote synthesis and release of cortisol, the primary glucocorticoid involved in the systemic stress response [64]. Cortisol, a steroid hormone and central component of the HPA axis, is synthesized from cholesterol in the zona fasciculata and reticularis of the adrenal cortex. Under normal physiologic conditions, activation of the HPA axis promotes adaptive responses to acute stress through tightly regulated glucocorticoid signaling [65]. Through negative feedback mechanisms, cortisol normally suppresses additional CRH and ACTH release, thereby restoring physiologic equilibrium.

Although transient cortisol release may be protective by modulating inflammatory responses and promoting anti-inflammatory signaling, chronic or repeated stress exposure can lead to persistent HPA axis activation and impaired feedback regulation [66]. Cortisol receptors include glucocorticoid receptors (GRs) and mineralocorticoid receptors, located throughout the CNS and limbic systems, respectively [67]. Cortisol binds to cytoplasmic GRs, resulting in translocation of the cortisol–GR complex to the nucleus. Activated GRs may function as monomers or homodimers, which differentially regulate gene transcription. In particular, GR homodimers promote transcription of anti-inflammatory proteins including annexin I, IκBα, and MAPK phosphatase [68,69,70]. In contrast, GR monomers can suppress proinflammatory signaling pathways through inhibition of transcription factors such as NF-κB, reducing inflammatory gene expression [68,71]. Prolonged glucocorticoid exposure is associated with GR desensitization, dysregulated cortisol signaling, and maladaptive neuroendocrine responses.

Glucocorticoid signaling also exerts suppressive effects on neurotrophin expression. Prolonged cortisol elevation downregulates brain-derived neurotrophic factor (BDNF) expression through glucocorticoid receptor-mediated repression of BDNF gene transcription, particularly in the hippocampus and prefrontal cortex [72]. BDNF is essential for neuronal survival, synaptic plasticity, and hippocampal neurogenesis, and its suppression by chronic glucocorticoid exposure represents an important biomolecular mechanism linking HPA axis dysregulation to structural brain changes and cognitive vulnerability [72,73]. Genetic variation at the BDNF rs6265 single nucleotide polymorphism (SNP), commonly referred to as the Val66Met polymorphism, further modulates this vulnerability. Individuals carrying the A (Met) allele exhibit impaired activity-dependent BDNF secretion and greater susceptibility to stress-induced hippocampal atrophy compared with G (Val) homozygotes [74].

Emerging evidence links perioperative HPA axis activation to postoperative neurocognitive disorders; specifically, surgical stress in aged animal models induces glucocorticoid receptor phosphorylation, reduces hippocampal BDNF expression, and exacerbates cognitive dysfunction, suggesting that excessive glucocorticoid signaling may directly contribute to cognitive decline [5]. Consistent with these experimental findings, prospective clinical studies have demonstrated that elevated postoperative serum cortisol concentrations are independently associated with an increased risk of both postoperative cognitive dysfunction and postoperative delirium following coronary artery bypass graft surgery [75,76]. Together, these findings provide translational evidence supporting HPA axis dysregulation as a mechanistic link between chronic stress sensitization and postoperative neurocognitive disorders.

Collectively, these findings suggest that chronic HPA axis dysregulation represents an important biological mechanism through which mood disorders may increase vulnerability to perioperative neurocognitive disorders. Persistent glucocorticoid dysregulation, impaired BDNF signaling, and exaggerated stress responses may prime the CNS for an amplified neuroinflammatory response following surgical stress, thereby reducing neurocognitive resilience in the perioperative period. Although accumulating preclinical and prospective clinical evidence supports this relationship, the relative contribution of HPA axis dysfunction to postoperative cognitive outcomes remains incompletely understood. Additional prospective studies integrating perioperative cortisol measurements, molecular biomarkers, and longitudinal neurocognitive assessments are needed to determine whether HPA axis dysregulation independently predicts PND risk and represents a viable therapeutic target.

### 3.5. Tryptophan–Kynurenine Pathway Dysregulation

The tryptophan–kynurenine pathway is an important biomolecular mechanism linking peripheral inflammatory signaling to central neurotoxicity and cognitive vulnerability. Under homeostatic conditions, tryptophan is primarily metabolized along the serotonergic pathway, contributing to the biosynthesis of serotonin and melatonin [77,78]. However, during states of immune activation and elevated inflammatory cytokine signaling, tryptophan catabolism is preferentially shunted toward the kynurenine pathway through upregulation of the rate-limiting enzyme indoleamine 2,3-dioxygenase-1 (IDO-1) [77,79].

IDO-1 is expressed in peripheral immune cells, microglia, and astrocytes, and its expression is tightly regulated by proinflammatory mediators including IFN-γ, TNF-α, and IL-6 (cytokines were consistently elevated following surgical trauma and in patients with MDD) [79,80]. IDO-1 activation catalyzes the conversion of tryptophan to kynurenine, which is subsequently metabolized along two enzymatic pathways with opposing neuroactive properties [78,80]. In the neurotoxic branch, kynurenine is converted by kynurenine monooxygenase (KMO) to 3-hydroxykynurenine and ultimately to quinolinic acid (QUIN), an N-methyl-D-aspartate (NMDA) receptor agonist [78,80]. Excessive QUIN accumulation promotes excitotoxic neuronal injury, oxidative stress, and disruption of hippocampal synaptic plasticity [78]. In the neuroprotective branch, kynurenine aminotransferases (KATs) convert kynurenine to kynurenic acid (KYNA), an NMDA receptor antagonist with anti-excitotoxic properties [78]. Neuronal vulnerability is therefore affected by the balance between these two branches.

Inflammatory conditions associated with surgical stress and mood disorders preferentially drive tryptophan metabolism toward the neurotoxic KMO branch, increasing the QUIN:KYNA ratio and promoting excitotoxic signaling within regions important for cognition, particularly the hippocampus and prefrontal cortex [81,82]. Concurrently, IDO-1-mediated tryptophan depletion reduces substrate availability for serotonin synthesis, further disrupting monoaminergic signaling and contributing to the neurochemical abnormalities characteristic of MDD [81,83]. Elevated kynurenine pathway metabolites, including QUIN and the QUIN:KYNA ratio, have been documented in patients with MDD and are associated with anhedonia, cognitive impairment, and treatment resistance [83,84].

Increasing evidence suggests kynurenine pathway dysregulation may also contribute to the development of PNDs [85]. In the perioperative setting, surgery-induced inflammation further amplifies IDO activation, promoting accumulation of neurotoxic kynurenine metabolites at the expense of neuroprotective pathways. This imbalance leads to accumulation of neuroactive kynurenine metabolites that have been associated with postoperative cognitive dysfunction through enhanced neuroinflammation, excitotoxicity, and impaired synaptic plasticity. Recent experimental work further demonstrated that anesthesia and surgery increase intestinal IDO activity through interferon-γ-producing innate lymphoid cells, resulting in elevated kynurenine levels and postoperative cognitive impairment in aged mice, with pharmacologic inhibition of the IDO–kynurenine pathway showing attenuation of these cognitive deficits [85,86].

HPA axis dysregulation further amplifies kynurenine pathway activation. Elevated glucocorticoids upregulate tryptophan 2,3-dioxygenase (TDO), a hepatic enzyme that also catalyzes tryptophan to kynurenine conversion, providing an additional cortisol-dependent mechanism through which chronic stress and mood disorders may augment neurotoxic kynurenine metabolism [87]. This glucocorticoid–IDO-1/TDO interaction creates a self-reinforcing cycle in which HPA axis dysregulation and peripheral inflammation converge to amplify central neurotoxic signaling.

In the perioperative context, surgical trauma-induced cytokine release acutely activates IDO-1, driving rapid tryptophan depletion and increased QUIN production while the CNS may already be sensitized by preexisting mood disorder-related kynurenine dysregulation [85,88]. This perioperative amplification of neurotoxic kynurenine metabolism may represent an important biomolecular mechanism contributing to perioperative neurocognitive disorders, particularly in patients with preexisting mood disorders characterized by chronic IDO-1 activation and a reduced KYNA:QUIN ratio [89]. Direct evidence demonstrating that perioperative modulation of the kynurenine pathway improves neurocognitive outcomes is currently lacking, making this an important area for future investigation.

## 4. Mood Disorders as Preoperative Vulnerability States

**Figure 4:**
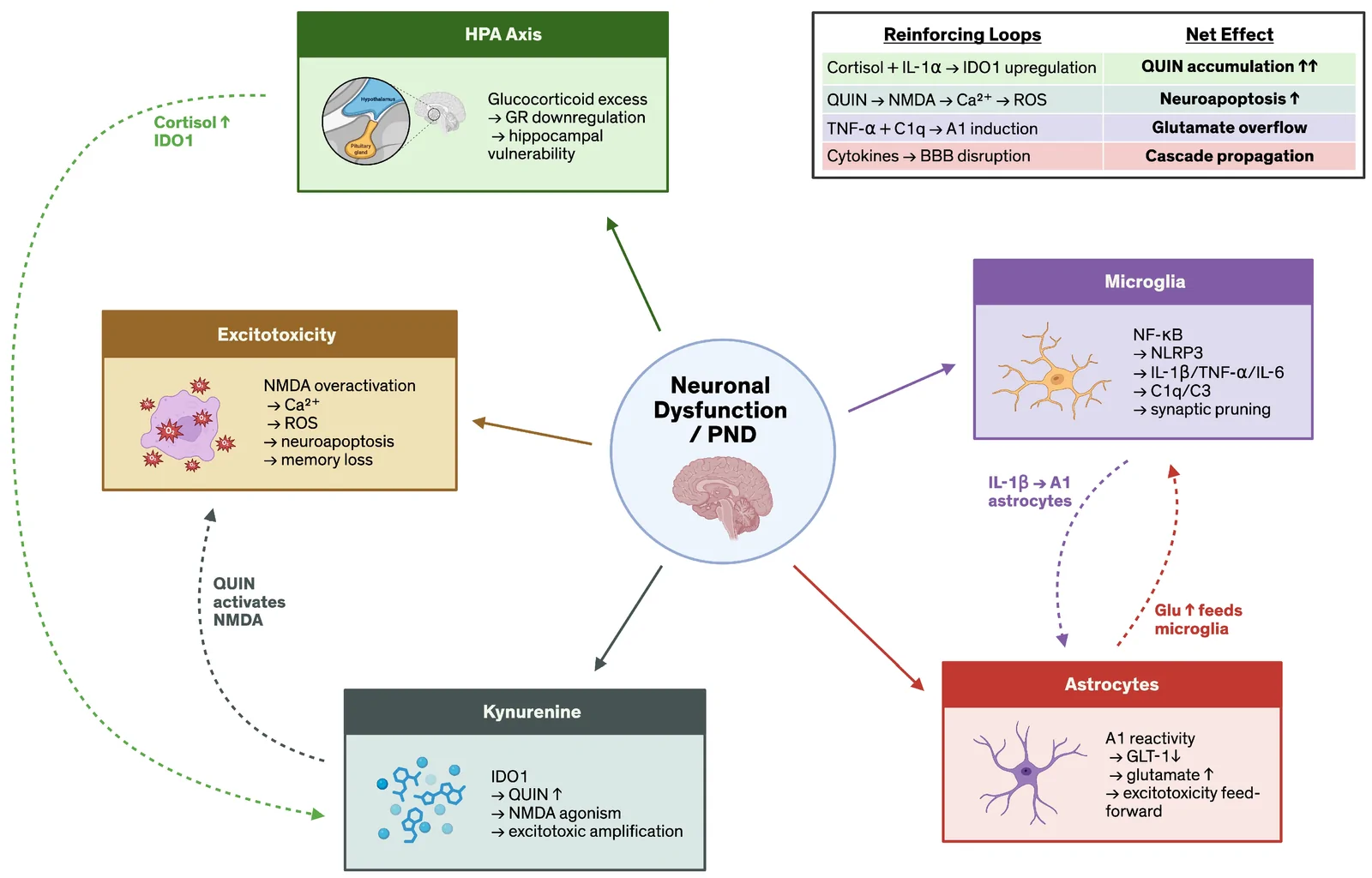
Integrated mechanisms linking mood disorders to perioperative neurocognitive disorders. Created in BioRender https://BioRender.com/umt4we8, accessed on 25 June 2026.

Mood disorders are increasingly recognized as systemic disorders involving chronic neuroinflammation, neuroendocrine dysregulation, and impaired neuroplasticity rather than isolated disturbances in neurotransmitter signaling alone (Figure 4). Major depressive disorder and bipolar disorder share substantial mechanistic overlap with pathways implicated in PND, including HPA axis dysfunction, microglial activation, oxidative stress, BBB disruption, and altered neurotransmission [26,27,31,33]. These overlapping mechanisms suggest that patients with preexisting mood disorders enter the perioperative period in a biologically sensitized state that increases vulnerability to exaggerated neuroinflammatory responses and postoperative cognitive dysfunction.

### 4.1. Major Depressive Disorder (MDD)

The pathophysiology of MDD is understood to involve a combination of neurotransmitter dysfunction, stress-response dysregulation, neuroimmune activation, and impaired neuroplasticity. Although dysregulation of monoaminergic signaling, particularly involving serotonin, dopamine, and norepinephrine, remains central to current pharmacologic therapies, growing evidence suggests that neuroinflammatory and neuroendocrine abnormalities play equally important roles in disease pathogenesis [26].

Chronic stress can drive HPA axis dysfunction leading to MDD, but MDD itself can further dysregulate the stress response, creating a reinforcing cycle. In MDD, persistent activation of the HPA axis can lead to prolonged glucocorticoid exposure, glucocorticoid receptor desensitization, and impaired negative feedback regulation [90]. Sustained cortisol elevation contributes to a proinflammatory environment characterized by increased cytokine signaling, astrocyte and microglial activation, and disruption of neuronal homeostasis. Elevated concentrations of inflammatory mediators including IL-1β, IL-6, and TNF-α have consistently been identified in patients with MDD, further supporting the role of chronic neuroinflammation in depressive symptomatology [91,92].

The emerging literature suggests that astrocyte dysfunction plays a role in MDD pathophysiology through chronic stress responses inducing structural and functional changes in astrocytes [93]. In particular, studies have demonstrated reduced astrocyte density in several brain regions implicated in mood regulation, including the prefrontal cortex and hippocampus [94,95]. At the same time, region-specific morphological changes have been observed, including enlargement of astrocyte cell bodies within the white matter of the anterior cingulate cortex in postmortem studies of individuals with MDD who died by suicide [96]. A central component of astrocytes is their role in regulating the BBB through their astrocytic foot processes. One study found a 50% reduction in astrocytic foot process coverage of blood vessels in subjects who had MDD compared to those who did not [97]. These findings suggest that chronic stress and depressive states may disrupt normal astrocyte homeostasis and function, impairing their ability to regulate neurotransmitter signaling, provide metabolic support to neurons, and maintain synaptic plasticity. Additionally, stress-induced astrocyte dysfunction may promote a reactive phenotype characterized by increased inflammatory signaling and altered crosstalk with microglia, ultimately establishing a primed neuroimmune state.

Persistent neuroinflammatory signaling in MDD has also been associated with structural and functional alterations within brain regions critical for cognition and emotional regulation, particularly the hippocampus and prefrontal cortex [98]. These changes may impair memory formation and increase susceptibility to cognitive dysfunction in the perioperative setting. Animal models have been essential for understanding HPA axis function on depressive-like symptoms. One study found that repeated, but not acute, corticosterone injections lead to depressive-like behaviors in rats, highlighting the detrimental effects of sustained cortisol elevation on mood regulation [99]. Animal models have also elicited important biomarkers related to depression-like behavior such as IL-1β and TNF-α [100,101]. Other studies have characterized the impact of chronic stress on long-term synaptic plasticity in the rat hippocampus, suggesting chronic stress may have impacts on future cognitive functioning [102].

These inflammatory and neuroendocrine abnormalities overlap substantially with pathways implicated in perioperative neuroinflammation, positioning patients with MDD in a pre-sensitized neuroimmune state prior to surgery (Figure 4).

Beyond shared biological mechanisms, there is growing clinical evidence that supports MDD as an independent risk for PND. A recent systematic review involving more than 42 studies with more than 4.6 million patients demonstrated that individuals with preoperative depression had nearly twice the risk of developing postoperative delirium compared with patients without depression [103]. Similarly, an earlier meta-analysis of cardiac surgery patients reported that preoperative depression was associated with more than a twofold increase in the odds of postoperative delirium [104]. These clinical observations support the hypothesis that chronic neuroimmune activation, HPA axis dysregulation, impaired synaptic plasticity, and reduced cognitive reserve in MDD create a pre-sensitized state that lowers the threshold for exaggerated neuroinflammatory responses following surgical stress.

### 4.2. Bipolar Disorder

Emerging evidence links bipolar disorder to an increased vulnerability for PNDs via overlapping neuroinflammatory and neuroendocrine mechanisms, although direct evaluations of PNDs in bipolar surgical cohorts remain limited.

HPA axis dysregulation represents a major point of mechanistic overlap. Patients with bipolar disorder frequently exhibit elevated cortisol levels, impaired feedback inhibition, and abnormal ACTH signaling, particularly during mood episodes [105]. Acute surgical stress independently activates the HPA axis and inflammatory signaling pathways, thereby compounding preexisting neuroendocrine dysfunction and directly increasing neuronal vulnerability [106].

Bipolar disorder is also characterized by dysregulation of dopaminergic, serotonergic, noradrenergic, and GABA signaling, contributing to impaired neurochemical homeostasis and vulnerability to cognitive instability [107,108]. Oxidative stress, mitochondrial dysfunction, and chronic inflammation further contribute to disruption of tight junction proteins (e.g., claudin-5), compromising neurovascular unit integrity and increasing BBB permeability [109].

Postmortem studies have reported region-specific alterations in microglial morphology and activation markers in bipolar disorder, though findings have been inconsistent across studies [110]. Peripheral cytokine profiling has demonstrated state-dependent elevations in IL-6, IL-1β, and TNF-α, with particularly pronounced increases during manic and depressive episodes compared to euthymic periods [111]. These cytokine fluctuations suggest that neuroimmune dysregulation in bipolar disorder is episodically amplified, potentially compounding perioperative inflammatory responses in patients whose illness is insufficiently controlled or who experience mood episodes near the time of surgery.

Mitochondrial dysfunction is another potential cause of biomolecular vulnerability in bipolar disorder, with converging evidence implicating impaired electron transport chain function at complex I, resulting in reduced ATP synthesis, increased superoxide generation, and elevated oxidative stress burden [112]. Mitochondrial DNA abnormalities, including deletions and copy number variations, have also been reported and are thought to compromise mitochondrial biogenesis and respiratory efficiency [112]. These deficits are particularly consequential in neurons, which are uniquely dependent on oxidative phosphorylation for maintenance of membrane potential, synaptic transmission, and calcium homeostasis [113]. The baseline reduction in mitochondrial reserve capacity in patients with bipolar disorder directly impairs neuronal resilience against the secondary oxidative burden generated by surgical stress.

Neurotrophin signaling abnormalities further contribute to the vulnerability profile of bipolar disorder. Reduced BDNF levels have been documented across mood episodes, with state-dependent fluctuations reflecting episodic HPA axis dysregulation and inflammatory signaling [114]. The BDNF Val66Met polymorphism has additionally been associated with increased susceptibility to bipolar disorder and greater cognitive impairment across memory and executive function domains [114]. Baseline deficits in BDNF-mediated neuroprotection impair neuronal resilience against acute surgical stress, amplifying the convergent impact of HPA axis dysregulation and neuroinflammation on postoperative cognitive decline.

Sleep and circadian dysregulation may additionally exacerbate vulnerability. Bipolar disorder is characterized by persistent sleep disturbances and reduced melatonin signaling, even during euthymic periods [115]. Sleep disruption is also strongly associated with delirium in perioperative and critical care settings. The depletion of melatonin exacerbates HPA axis dysregulation, oxidative stress, and neuroinflammation, which directly intersects with dopaminergic pathways to solidify a cycle of neurobiological instability.

The convergence of HPA axis dysregulation, state-dependent neuroimmune activation, mitochondrial dysfunction, oxidative stress, BBB disruption, and impaired neurotrophin signaling establishes patients with bipolar disorder as a highly vulnerable perioperative population (Figure 4). In these individuals, preexisting multi-domain neurobiological instability lowers the threshold for exaggerated neuroinflammatory responses, postoperative cognitive dysfunction, and prolonged neurocognitive recovery.

## 5. Perioperative Implications and Future Directions

### 5.1. Anesthetic Considerations

Because neuroinflammation, microglial activation, and stress-response dysregulation drive perioperative neurocognitive disorders (PNDs), anesthetic management directly modulates neuroimmune signaling pathways to shape neurocognitive outcomes. Investigating the depth of anesthesia yields divergent clinical evidence; one meta-analysis demonstrated that higher bispectral index (BIS) values correlate with reduced POCD and cognitive decline at three months, indicating that deeper anesthetic planes exacerbate PND risk [116]. Conversely, a randomized controlled study of elderly patients undergoing abdominal surgery found that BIS-guided deep anesthesia reduced POCD at one week and attenuated peripheral inflammatory markers, although this neurocognitive benefit was no longer evident at three months [117].

The specific anesthetic regimen—particularly total intravenous anesthesia (TIVA) versus inhalational-based techniques—simultaneously alters perioperative inflammatory cascades. While certain meta-analytic data associate TIVA with a lower incidence of POCD beyond thirty days compared to volatile agents [118], competing analyses offer only low-certainty evidence regarding TIVA’s capacity to attenuate POCD or reduce hospital length of stay. Among anesthetic adjuncts, dexmedetomidine has emerged as a potential strategy for mitigating PNDs due to its anti-inflammatory and neuroprotective effects. A recent meta-analysis found that dexmedetomidine was associated with a reduced incidence of postoperative delirium; however, this benefit was no longer statistically significant after exclusion of studies at high risk of bias, highlighting the need for additional high-quality randomized trials to clarify its role in perioperative neuroprotection [119]. This clinical heterogeneity underscores an evolving paradigm in anesthetic optimization strategies designed to mitigate postoperative neurocognitive decline.

### 5.2. Pain Management

Perioperative pain management directly dictates a patient’s psychological recovery and physical trajectory. Inadequate postoperative pain control precipitates a cascade of adverse systemic events, including delayed recovery, pneumonia, sleep fragmentation, and profound hypercortisolemia. Crucially, both uncontrolled pain and excessive opioid administration independently drive adverse perioperative neurocognitive outcomes, specifically postoperative delirium (POD) and postoperative cognitive dysfunction (POCD). Multimodal analgesic strategies represent the definitive first-line standard to attenuate pain severity while minimizing opioid dependency [120]. In older or medically complex populations, opioid exposure has been associated with increased risk of neurocognitive decline by inducing sedation, respiratory depression, and severe architectural sleep disruption [121]. Consequently, optimized multimodal analgesia serves as a critical clinical intervention to mitigate perioperative neurocognitive risks.

Minimizing physiological stress and excessive neuroinflammatory activation through targeted perioperative strategies directly reduces postoperative cognitive and affective complications in vulnerable populations, such as patients with preexisting mood disorders.

## 6. Conclusions

“Perioperative neurocognitive disorders” (PNDs) is a term that has become more popular in recent years as it describes a broad understanding of the cognitive changes that occur around surgery. The transition to using PNDs reflects an evolution in understanding the cognitive changes that can occur at any stage of surgery and allows us to identify underlying etiologies of PNDs as well as understand and optimize clinical outcomes for patients undergoing surgery.

Emerging evidence suggests that mood disorders such as MDD and bipolar disorder share mechanistic overlap with pathways implicated in PNDs, including chronic neuroinflammation, HPA axis dysfunction, oxidative stress, BBB disruption, and astrocyte and microglial activation. In individuals with mood disorders, persistent inflammatory signaling and chronic stress exposure may promote a primed state characterized by astrocyte and microglial sensitization, impaired stress-response regulation, and altered synaptic plasticity, ultimately creating a heightened baseline vulnerability to perioperative neurocognitive dysfunction.

Transitioning the clinical perception of mood disorders to recognize them as critical perioperative vulnerability states holds profound implications for preoperative optimization and the preservation of functional, cognitive, and behavioral recovery. Delineating the overlapping neuroinflammatory and neuroendocrine pathways linking mood disorders and PNDs facilitates the early identification of high-risk cohorts, refines perioperative risk stratification, and drives the development of targeted interventions designed to modulate neuroimmune activation and preserve neuronal homeostasis.

Although growing evidence supports mechanistic overlap between mood disorders and PNDs, much of the mechanistic evidence is derived from preclinical and postmortem studies and observational clinical investigations, limiting definitive conclusions regarding causality. Furthermore, the relative contribution of individual pathways—including HPA axis dysregulation, microglial and astrocyte dysfunction, oxidative stress, and kynurenine pathway dysregulation—to postoperative cognitive outcomes has yet to be fully established.

Future research should prioritize prospective clinical studies to determine whether preexisting mood disorders independently increase the risk of PNDs after adjustment for established perioperative risk factors. Longitudinal investigations integrating perioperative cognitive assessments with biomarkers of neuroinflammation, HPA axis activity, glial activation, and kynurenine metabolism will be essential to validate the mechanistic pathways proposed in this review. In addition, randomized clinical trials evaluating targeted perioperative interventions—including optimization of mood disorders before surgery, opioid-sparing analgesic strategies, individualized anesthetic management, and neuroprotective anesthetic adjuncts such as dexmedetomidine—are needed to determine whether modulation of these pathways can improve postoperative neurocognitive outcomes.

As the mechanistic understanding of PNDs evolves, integrating psychiatric, neuroimmune, and neuroendocrine frameworks may improve perioperative risk stratification, facilitate the development of personalized management strategies, and ultimately reduce postoperative cognitive complications while improving long-term functional recovery and quality of life.

## Data Availability

No new data were created or analyzed in this study. Data sharing is not applicable to this article.
